# The Growth and Tumor Suppressors NORE1A and RASSF1A Are Targets for Calpain-Mediated Proteolysis

**DOI:** 10.1371/journal.pone.0003997

**Published:** 2008-12-22

**Authors:** Sergey Kuznetsov, Andrei V. Khokhlatchev

**Affiliations:** 1 Department of Physics, University of Rhode Island, East Hall, Kingston, Rhode Island, United States of America; 2 Department of Pathology, University of Virginia, Charlottesville, Virginia, United States of America; Ordway Research Institute, United States of America

## Abstract

**Background:**

NORE1A and RASSF1A are growth and tumour suppressors inactivated in a variety of cancers. Methylation of NORE1A and RASSF1A promoters is the predominant mechanism for downregulation of these proteins; however, other mechanisms are likely to exist.

**Methodology/Principal Findings:**

Here we describe a proteolysis of NORE1A and RASSF1A by calpains as alternative mechanism of their downregulation. Extracts of H358 cell line, a human bronchoalveolar carcinoma, and H460, a large cell carcinoma, were capable of proteolysis of NORE1A protein in the calpain-dependent manner. Likewise, RASSF1A tumor suppressor was proteolyzed by the H358 cell extract. Addition of calpain inhibitor to H358 and H460 cells growing in tissue culture resulted in re-expression of endogenous NORE1A. A survey of 10 human lung tumours revealed that three of them contain an activity capable of inducing NORE1A degradation.

**Conclusions/Significance:**

Thus, degradation by calpains is a novel mechanism for downregulation of NORE1A and RASSF1A proteins and might be the mechanism allowing cancer cells to escape growth suppression.

## Introduction

The NORE1A protein was identified in a yeast two-hybrid screen as a putative Ras effector that binds Ras protein in a GTP-dependent manner [1]. The full-length NORE1A cDNA encodes a 47-kDa basic protein that has a proline-rich N-terminus and a cysteine-rich domain that is homologous to the C1 domains of PKC and Raf. Its Ras-association (RA) domain is located centrally [2]. In contrast to the well-known Ras pathways promoting proliferation and oncogenesis, NORE1A mediates growth suppression. NORE1A is expressed in most normal tissues but is lost in cancer (see [3] for review). NORE1A downregulation in cancer appears to be due to hypermethylation of its promoter CpG islands [4], [5]. Reconstitution of NORE1A expression induces growth arrest as well as cell death in a variety of tumor cell lines [6]–[8].

Ras-association domain family 1 (RASSF1) was discovered as a tumor suppressor gene located on human chromosome 3p21 in a segment that is deleted in many human solid tumors [9]. Expression of the longest splice isoform of the *RASSF1* gene, RASSF1A, is downregulated by selective hypermethylation of its promoter CpG islands in at least 37 tumour types, according to the recent review [3]. *RASSF1A* is thought to be the most frequently methylated gene described in human cancers so far [10]. RASSF1A is the closest relative to NORE1A (41% identity at the amino acid level); it is also capable of binding to activated Ras [2]. Re-expression of RASSF1A in various tumour cell lines where this gene was deleted or its promoter is methylated inhibits cell growth, invasion, stimulates apoptosis and reduces tumorigenicity in mouse models [11], [12]. Targeted disruption of the RASSF1A gene increases spontaneous tumorigenesis. The exposure of RASSF1A-null mice to physical and chemical mutagens and carcinogens increased tumour susceptibility relative to controls [13], [14].

Although the promoter methylation is apparently the major mechanism of silencing of NORE1A and RASSF1A expression, other mechanisms likely exists. NORE1A expresses in human adrenal medulla while its expression was lost in pheochromocytoma and abdominal paraganglioma tumors. The NORE1A promoter in these tumors was not methylated but no mRNA expression was detected. In addition, both NORE1A mRNA and protein levels are severely downregulated in follicular thyroid carcinomas harboring a PAX8-PPARγ translocation; however, the NORE1A promoter was not methylated [15]. Recent studies suggested that up to 15% of tumors may contain inactivating point mutations in RASSF1A [16].

In the current study, we describe that NORE1A and RASSF1A proteins undergo a proteolytic cleavage by an activity present in extracts of several human tumor cell lines. This proteolytic activity was sensitive to inhibitors of proteases called calpains. A survey of 10 human lung cancer samples revealed that at least three of them also contains an activity capable of proteolyzing NORE1A. Thus, calpain-mediated degradation could be a novel mechanism of inactivation NORE1A and RASSF1A in cancers.

## Results

### NORE1A and RASSF1A proteins are proteolyzed by an activity present in extract of some human tumor cell lines

**Figure 1:**
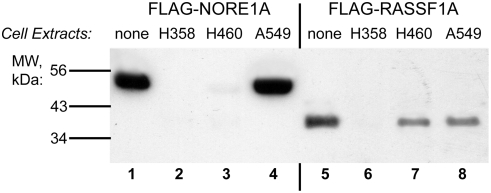
Cleavage of NORE1A and RASSF1A by an activity expressed in some tumor cell lines. Full-length NORE1A and RASSF1A, tagged at the N-terminus with the FLAG tag, were expressed in HEK293 cells, immunopurified on FLAG beads and beads were eluted with FLAG-peptide. NORE1A (lanes 1–4) or RASSF1A (lanes 5–8) were incubated with the Buffer A (lanes 1, 5) or H358 cell lysate (lanes 2, 6), or H460 cell lysates (lanes 3, 7), or A549 cell lysates (lanes 4, 8) for 30 minutes at 37°C. After incubation, samples were subjected to gel electrophoresis followed by Western blotting with anti-FLAG antibodies.

We found that incubation of NORE1A protein for 30 minutes at 37°C with extracts of human lung cancer cell lines H358 (bronchoalveolar carcinoma) and H460 (large cell carcinoma) resulted in proteolysis of the NORE1A protein (Figure 1, lanes 2 and 3). The RASSF1A tumor suppressor was proteolyzed by the H358 cell extract (Fig. 1, lane 6) but not H460 cell extract (Fig. 1, lane 7). Cell extracts of A549 lung adenocarcinoma cells (Fig. 1, lanes 4 and 8), normal human bronchial cells, normal human fibroblasts, HEK293 cells, H157 squamous cell carcinoma and SW1573 lung alveolar carcinoma cells (data not shown) were devoid of this proteolytic activity.

### Mapping of cleavage sites within NORE1A

**Figure 2:**
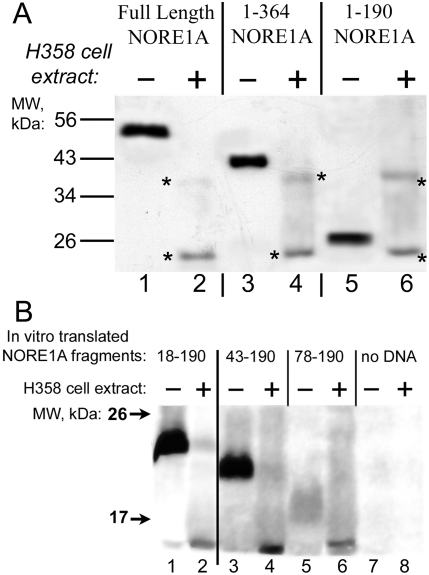
NORE1A cleavage occurs at the N-terminus of the protein. A: Full-length NORE1A (lanes 1, 2) or its fragments aa 1–364 (lanes 3, 4) or aa 1–190 (lanes 5, 6), tagged N-terminally with the FLAG tag, immunopurified as described in Figure 1, were incubated with the lysis buffer (lanes 1, 3, 5) or with H358 cell lysate (lanes 2, 4, 6) for 30 minutes at 37°C. After incubation, samples were subjected to gel electrophoresis followed by Western blotting with anti-FLAG antibodies. Asterisks denote non-specific bands. B: NORE1A fragments, amino acids 18–190, 43–190 and 78–190 were synthesized in vitro using Promega TnT T7 Quick for PCR DNA kit in the presence of ^35^S-Methionine. Each fragment contained an additional methionine as start codon and two extra methionines at the C-terminus to facilitate detection. An aliquot of translation mixture was incubated with 4× excess (v/v) of H358 cell extract or with Buffer A for 1 hour at 37°C, resolved on SDS gel and transferred to Immobilon. Shown is the autoradiogram of the membrane obtained by Phosphoimager.

Experiments with the proteolysis of NORE1A fragments suggested that it might occur within the first 190 amino acids of the protein (Figure 2A). This region contains Proline-rich region (aa 1–119) and the Cysteine-rich C1 domain (aa 120–166) potentially involved in lipid binding [17]. Attempts to map the cleavage site(s) more precisely suggested that the cleavage occurs after position 78 in NORE1A in the Proline-rich region, since NORE1A fragment 78–190 is cleaved by H358 cell extract (Figure 2B, lane 6).

### Cleavage of NORE1A and RASSF1A by tumor cell extracts requires calcium ions and is prevented by a calpain inhibitor

**Figure 3:**
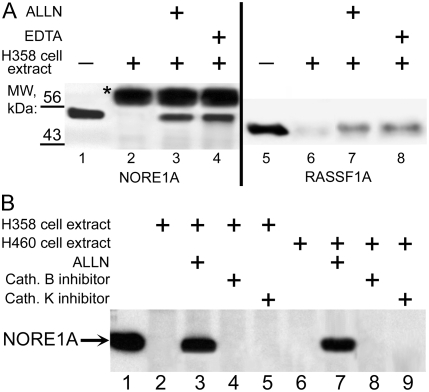
A: Cleavage of NORE1A and RASSF1A requires calcium and is sensitive to calpain inhibitors. NORE1A, tagged N-terminally with the FLAG tag, was expressed in HEK293 cells and adsorbed on FLAG beads (lanes 1–4). RASSF1A, tagged N-terminally with the FLAG tag, was expressed as described in Figure 1 (lanes 5–8). Equal amount of beads containing NORE1A, or RASSF1A were incubated for 30 min at 37°C with H358 cells extract with no additions (lanes 2, 6) or with 1 µM of calpain inhibitor ALLN (lanes 3, 7) or with 50 mM EDTA (lanes 4, 8). NORE1A and RASSF1A, eluted from beads with the FLAG peptide without cleavage, was used as controls (lanes 1 and 5, respectively). After incubation, NORE1A-containing beads were extensively washed. Samples were subjected to gel electrophoresis followed by Western blotting with anti-FLAG antibodies. Asterisk denoted IgG heavy chain eluted from FLAG beads. B, Cathepsin B and Cathepsin K are not responsible for NORE1A cleavage by H358 and H460 cell lysates: NORE1A, tagged N-terminally with the FLAG tag, was expressed as described in Methods and adsorbed on FLAG beads. Equal amounts of NORE1A were incubated for 30 min at 37°C with H358 cells extract (lanes 2–5) or H460 cell extract (lanes 6–9) with no additions (lanes 2, 6) or with ALLN (1 µM, lanes 3, 7) or with inhibitor of Cathepsin B (10 µM, lanes 4, 8) or with inhibitor of Cathepsin K (2 µM, lanes 5, 9). NORE1A eluted from beads with the FLAG peptide was used as control (lane 1). After incubation, beads were extensively washed and proteins retained on them were subjected to gel electrophoresis followed by Western blotting with anti-FLAG antibodies.

Since the N-terminus of NORE1A is reach in prolines, we hypothesized that calpains, a class of proteases which have preference to Proline-rich sequences, might be responsible for its cleavage. Indeed, addition of a broad-spectrum calpain inhibitor, ALLN (N-Acetyl-Leu-Leu-Nle-CHO), to the extract of H358 cells completely inhibited NORE1A (Figure 3A, lane 3) and RASSF1A (Figure 3A, lane 7) proteolysis.

Calpains are calcium-dependent proteases. As shown on Figure 3A, lanes 4 and 8, chelation of calcium ions by addition of EDTA inhibited cleavage of NORE1A and RASSF1A by H358 cell lysate, consisting with the hypothesis that calpains are responsible for the cleavage.

The ALLN compound is not specific for calpains: it could also inhibit Cathepsin B and Cathepsin K proteases. However, as shown on Figure 3B, the addition of Cathepsin B and Cathepsin K inhibitors did not prevent NORE1 cleavage by lysates of H358 (lanes 4, 5) and H460 (lanes 8, 9) cells. The requirement of Ca^2+^ ions for NORE1A and RASSF1A cleavage also argues against cathepsins B and K involvement, since they are not calcium-dependent proteases.

### Inhibition of calpain in cultured H358 and H460 tumor cells results in re-expression of NORE1A protein

**Figure 4:**
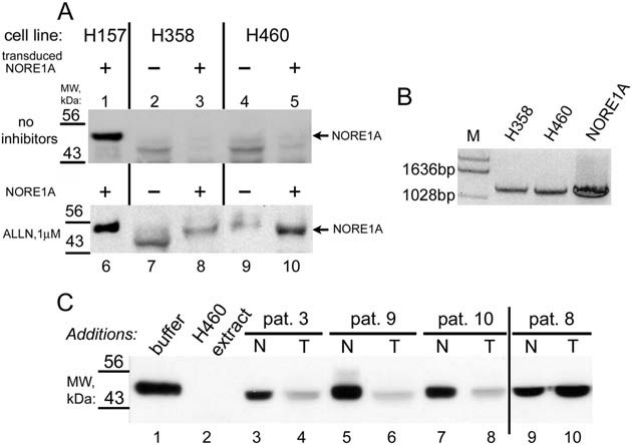
A, The addition of a calpain inhibitor ALLN to cultured cells prevents NORE1A degradation: NORE1A was re-expressed in H157 cells (lanes 1, 6), H358 cells (lanes 3, 8) and H460 cells (lanes 5, 10) using a vector with a drug-resistance marker with subsequent selection for the drug. Lanes 2, 7 represent H358 parental cells and lanes 4, 9 - H460 parental cells. Cells were cultured for 3 days without inhibitors (top panel) or with 1 µM ALLN (bottom panel). Cell extracts, equalized by α-tubulin, were probed with 10F10 antibody by Western Blotting. At 1 µM concentration ALLN did not induce apoptotic response in H358 and H460 cells (data not shown). B, NORE1A expression at the mRNA level in H358 and H460 cells: NORE1A cDNA was re-expressed in H358 and H460 cells using a vector with a drug-resistance marker. Total mRNA was extracted from pools of drug-resistant cells emerged after selection as indicated and analyzed by RT-PCR for NORE1A expression. Note that these cells did not express NORE1A protein (Fig 4A, lanes 3 and 5). NORE1A, cDNA of vector used for transduction was included as a positive control. M, DNA molecular weight markers. C: Extracts of human tumors are capable of degrading NORE1A. Human lung tumors from patients 3, 9, 10 and 8 and matching normal lung tissues were extracted into buffer for cell extraction and normalized by total amount of cellular protein. Equal amount of extracts from tumors (T) and normal tissues (N) were mixed with FLAG-tagged NORE1A, immunopurified as described in Methods. After thirty minute incubation at 37°C, electrophoretic sample buffer was added to 1× concentration, samples were boiled and subjected to Western blotting with anti-FLAG antibodies. As positive control for NORE1A degradation, NORE1A incubated with H460 cell extract (lane 2) was used.

NORE1A and RASSF1A are not expressed in H358 and H460 cells at the protein level [6], [8], [18]. Our attempts to stably express NORE1A protein in H358 and H460 cells using a retroviral or plasmid vector with the drug-resistance marker resulted in drug-resistant cell populations that have little or no expression of the full-length NORE1A protein detectable by Western blotting (Figure 4A, lanes 3 and 5). However, the NORE1A mRNA message was readily detected by reverse transcription-polymerase chain reaction (RT-PCR) in such drug-resistant cells (Fig. 4B). We suggested that the reason for this might be proteolysis of NORE1A by calpains in H358 and H460 cell lines. Indeed, when H358 and H460 cells transfected with NORE1A-expressing plasmid were propagated in tissue culture in the presence of a calpain inhibitor, a prominent NORE1A band appeared on Western blots (Figure 4A, lanes 8 and 10). Moreover, a weaker band with the mobility of NORE1A appeared in lysates of parental H358 and H460 cells, propagated in the presence of a calpain inhibitor, suggesting that inhibition of calpains resulted in re-expression of endogenous NORE1A in these lung cancer cell lines (Figure 4A, lanes 7 and 9).

### Extracts of some human tumors are capable of NORE1A cleavage

To determine whether human tumors may contain an activity that cleaves NORE1A, we examined extracts from ten lung cancers and matching non-tumor tissues form the same patient to cleave FLAG-NORE1A in vitro. As shown on Figure 4C, extracts from tumors taken from patients 3, 9 and 10 were capable of inducing NORE1A cleavage and degradation (lanes 4, 6 and 8) while extract from tumor taken from patient 8 (lane 10), as well as extracts taken from six other patients (data not shown) did not induce NORE1A cleavage. None of the extracts of normal tissues were capable of NORE1A cleavage (Figure 4C, lanes 3, 5, 7 and 9).

## Discussion

To the best of our knowledge, proteolysis of RASSF family tumor suppressors NORE1A and RASSF1A by calpains has not been described previously.

Calpains are family of calcium-dependent cysteine proteases. Several isoforms of mammalian calpains are known to date (calpains 1–16); the majority of them are ubiquitously expressed [19], [20]. Since NORE1A is proteolyzed by extract of two cell lines, H358 and H460, and RASSF1A is proteolyzed only by the extract of H358 cells, different calpain isoforms might be involved in proteolysis of NORE1A and RASSF1A.

Calpains are involved in regulatory cleavage of a number of substrates during cell adhesion, spreading, migration, differentiation, apoptosis and autophagy [19]–[21]. Calpains are also involved in regulation of cancer cell growth. Inhibition of calpain activity by cell-permeable inhibitors suppressed anchorage-independent growth of cells transformed by Src, Jun, Myc, Ki-Ras and Fos oncogenes [22]. However, in other studies calpain inhibition promotes anchorage-independent growth of NIH 3T3 cells [23]. Calpain activity was significantly higher in breast cancer samples relative to normal matching controls [24] and calpeptin, a cell-permeable specific inhibitor of calpain, inhibited growth of estrogen receptor-positive breast cancer cells [25].

Calpains are involved in cleavage and inactivation of tumor suppressors such as p53, its relative p73, NF2/merlin, and cyclin-dependent kinase inhibitors p19^INK4d^ [26] and p27^Kip1^ [27]–[30]. In tumor cells with wildtype p53, inhibition of calpain increased p53 level and enhanced apoptosis [31]. The Rb tumor suppressor is cleaved by calpain during tumor necrosis factor-alpha (TNFα)-induced apoptosis in HeLa cells. Calpain inhibitors stabilized Rb; mouse embryo fibroblasts with targeted disruption of a gene encoding calpain regulatory subunit CAPN4 have elevated Rb level and showed resistance to TNFα-induced apoptosis in comparison with wild-type cells [32]. The E7 transforming protein of the human papilloma virus (HPV) induces calpain-mediated Rb cleavage in vitro and in vivo; the cleaved Rb was deficient in inducing growth arrest in SAOS-2 cells. Calpain inhibitors interfered with the E7-mediated degradation of Rb and reduced the viability of HPV-transformed cells [33].

Our finding that activity capable of NORE1A proteolysis as found not only in cell lines but in human tumors argues against the hypothesis that calpain cleavage of NORE1A and perhaps RASSF1A is an artifact of tissue culture. The calpain-mediated cleavage of NORE1A and RASSF1 tumor suppressors might be a mechanism by which some human tumors escape growth and tumor suppression. Further studies will be needed to address the question whether inhibition of this cleavage might induce re-expression of NORE1A and RASSF1A proteins with subsequent growth arrest or cell death in tumor cells.

## Materials and Methods

### Cell Lines and Transfection

H358, H460, H157, HEK293 and A549 cells (ATCC, Manassas, VA) were cultured in high-glucose DMEM (Invitrogen, Carlsbad, CA) supplemented with 10% fetal bovine serum in a 5% CO_2_ atmosphere at 37°C. Plasmids encoding NORE1A or RASSF1A were transfected into 293 cells with Lipofectamine agent (Invitrogen) as described previously [8], [34].

### Reagents and Plasmids

Plasmids encoding NORE1A and RASSF1A tagged N-terminally with the FLAG tag were described previously [8]. All other constructs were prepared using standard molecular biology techniques and verified by sequencing. ALLN calpain inhibitor (cat # 208719), Cathepsin B inhibitor II (cat # 219385) and Cathepsin K inhibitor I (cat # 219377) were from EMD Biosciences. The monoclonal anti-NORE1A antibody 10F10 and all other reagents were previously described by Moshnikova et al. [8].

### Preparation of cell extracts and in vitro cleavage

Cells growing in tissue culture were extracted in the Buffer A: 30 mM HEPES, pH 7.4, 20 mM KCl, 1 mM NaVO_4_, 20 mM NaF, 20 mM β-glycerophosphate, 7.5 mM MgCl_2_, 1% Triton X-100, 2 mM EGTA, 0.1% 2-mercaptoethanol and protease inhibitor cocktail for mammalian cells and tissues, Sigma Cat # P8340 at 0.3% (v/v). Cell extracts were clarified by centrifugation at 16,100×g for 40 minutes at 4°C. Protein concentration was measured and aliquots of each extract containing equal amounts of protein was incubated with FLAG-tagged NORE1A or RASSF1A proteins expressed in 293 cells, purified on FLAG beads and eluted with 0.1 mg/ml of FLAG-peptide solution in Buffer A as described previously [8]. After thirty minute incubation at 37°C, electrophoretic sample buffer was added to 1× concentration, samples were boiled and subjected to Western blotting.

### Immunoprecipitation and Western Blot Analysis

Transfected 293 cells were lysed in buffer A and NORE1A and RASSF1A were immunoprecipitated from cell extract as described previously [8], [34]. In some experiments, NORE1A and RASSF1A were eluted from FLAG-beads as described above.

### Human Tumor samples

Anonymized specimens of human lung tumors and noncancerous tissue surrounding the tumor were procured using University of Virginia Biorepository and Tissue Research Facility. The procurement was done according to the institutional IRB policy. Tissues were extracted into buffer A and clarified by centrifugation and the extracts were used for in vitro cleavage.
